# Predictive value of stress hyperglycemia ratio for the occurrence of acute kidney injury in acute myocardial infarction patients with diabetes

**DOI:** 10.1186/s12872-021-01962-2

**Published:** 2021-03-30

**Authors:** Side Gao, Qingbo Liu, Hui Chen, Mengyue Yu, Hongwei Li

**Affiliations:** 1 Department of Cardiology, Cardiovascular Center, Beijing Friendship Hospital, Capital Medical University, Yongan Road 95, Xicheng District, 100050 Beijing China; 2 Department of Cardiology, Fuwai Hospital, National Center for Cardiovascular Diseases, Chinese Academy of Medical Sciences and Peking Union Medical College, Bei Li Shi Road 167, Xicheng District, 100037 Beijing China

**Keywords:** Stress hyperglycemia ratio, Diabetes, Acute myocardial infarction, Acute kidney injury, In-hospital outcomes

## Abstract

**Background:**

Acute hyperglycemia has been recognized as a robust predictor for occurrence of acute kidney injury (AKI) in nondiabetic patients with acute myocardial infarction (AMI), however, its discriminatory ability for AKI is unclear in diabetic patients after an AMI. Here, we investigated whether stress hyperglycemia ratio (SHR), a novel index with the combined evaluation of acute and chronic glycemic levels, may have a better predictive value of AKI as compared with admission glycemia alone in diabetic patients following AMI.

**Methods:**

SHR was calculated with admission blood glucose (ABG) divided by the glycated hemoglobin-derived estimated average glucose. A total of 1215 diabetic patients with AMI were enrolled and divided according to SHR tertiles. Baseline characteristics and outcomes were compared. The primary endpoint was AKI and secondary endpoints included all-cause death and cardiogenic shock during hospitalization. The logistic regression analysis was performed to identify potential risk factors. Accuracy was defined with area under the curve (AUC) by a receiver-operating characteristic (ROC) curve analysis.

**Results:**

In AMI patients with diabetes, the incidence of AKI (4.4%, 7.8%, 13.0%; *p* < 0.001), all-cause death (2.7%, 3.6%, 6.4%; *p* = 0.027) and cardiogenic shock (4.9%, 7.6%, 11.6%; *p* = 0.002) all increased with the rising tertile levels of SHR. After multivariate adjustment, elevated SHR was significantly associated with an increased risk of AKI (odds ratio 3.18, 95% confidence interval: 1.99–5.09, *p* < 0.001) while ABG was no longer a risk factor of AKI. The SHR was also strongly related to the AKI risk in subgroups of patients. At ROC analysis, SHR accurately predicted AKI in overall (AUC 0.64) and a risk model consisted of SHR, left ventricular ejection fraction, N-terminal B-type natriuretic peptide, and estimated glomerular filtration rate (eGFR) yielded a superior predictive value (AUC 0.83) for AKI.

**Conclusion:**

The novel index SHR is a better predictor of AKI and in-hospital mortality and morbidity than admission glycemia in AMI patients with diabetes.

**Supplementary Information:**

The online version contains supplementary material available at 10.1186/s12872-021-01962-2.

## Introduction

Acute kidney injury (AKI) is a frequent complication in patients with acute myocardial infarction (AMI) and remains a leading contributor to the poor prognosis even after optimal medication and revascularization with percutaneous coronary intervention (PCI) [1–4]. Among various risk factors for AKI, both diabetes mellitus (DM) and stress hyperglycemia have been emphasized for its good discrimination for AKI and cardiovascular (CV) adverse events [5–7]. Therefore, there is an urgent need to understand the critical role of elevated glycemia in AKI development and to establish an ideal biomarker for AKI prediction in AMI patients.

Elevated glycemia at admission has been used to identify stress hyperglycemia, however, its values are affected by both acute stress condition and chronic glycemic control, and a high value of admission blood glucose (ABG) doesn’t necessarily indicate an acute glucose-level rise in response to AMI, especially in DM patients with chronic glycemic elevation [8]. Previous studies also proved that the relationship between admission glycemia and the risk of AKI was prominent among nondiabetic patients, however, it was no longer significant in patients with DM [9, 10]. These findings suggest that a relative increase in glycemia may have more clinical implications in early recognition and prevention of AKI in AMI patients with DM. Recently, a novel index of stress hyperglycemia (stress hyperglycemia ratio, SHR) was proposed and defined as ABG divided by the estimated average glucose (eAG) [8], while eAG was derived from the glycated hemoglobin (HbA_1c_) [11]. Following its introduction, the performance of SHR has been developed and validated in AMI patients [12, 13], showing a superior discrimination for in-hospital morbidity and mortality than admission glycemia alone [14, 15]. Yet, data regarding the predictive value of SHR for AKI in AMI patients with DM are scarce. In the present study, we investigated whether the combined evaluation of acute and chronic glycemic levels as expressed by SHR could predict AKI in hospitalized AMI patients with DM and whether the predictive power of this ratio might be better than admission glycemia alone.

## Methods

### Study population

This was a single-center, retrospective and observational study, and patient data were retrieved from Cardiovascular Center Beijing Friendship Hospital Database (CBD BANK). A total of 1421 consecutive AMI patients with DM, both ST-segment-elevation myocardial infarction (STEMI) and non-ST-segment-elevation myocardial infarction (NSTEMI), were admitted to the Cardiac Care Unit at the cardiovascular center of Beijing Friendship Hospital between January 2013 and October 2017. We aimed to focus on patients who had a relatively stable creatinine level before and then developed AKI secondary to AMI, thus the following patients were excluded due to: (1) chronic renal failure need for regular hemodialysis (n = 11) or peritoneal dialysis (n = 5); (2) serum creatinine ≥ 442 μmol/L at first admission (n = 8); (3) concomitant with sepsis that may affect renal function (n = 4); (4) missing data of initial and peak creatinine values, admission glycemia and HbA_1c_ (n = 178). Final analysis was therefore performed on 1215 patients. All patients were treated with optimal medication according to current guidelines and recommendations [16], including aspirin, clopidogrel or ticagrelor, statin, angiotensin-converting enzyme inhibitor or angiotensin receptor blocker and β-blocker. These drugs were routinely prescribed since admission and were continued after discharge unless there were contraindications. Interventional procedures were performed and strategies were made at the expert operator’s discretion using standard techniques [17], including percutaneous transluminal coronary angioplasty, the second-generation drug eluting stent implantation, and the mechanical circulatory support with intra-aortic balloon pump (IABP). Adverse events during hospitalization were checked using medical records by a team of independent research physicians not involved in the treatment. This study was approved by the Ethics Committee of Beijing Friendship Hospital and was conducted in accordance with the Declaration of Helsinki. Written informed consent was obtained from all participants.

### Data collection

Baseline data on the demographic, clinical, laboratory and angiographic characteristics were obtained from in-person interviews and medical records. Admission blood glucose (ABG) was measured using standardized biochemical assay. HbA_1c_ was routinely tested with a high-performance liquid chromatography analyzer in hospitalized AMI patients, regardless of whether they had preexisting DM. As reported [11], the estimated average glucose (eAG) was derived from HbA_1c_ using the following equation: eAG(mmol/L) = [1.59 × HbA_1c_ (%) − 2.59]. The index SHR was calculated with the formula “ABG/eAG” in which ABG was divided by eAG, indicating a relative glycemic increase after correcting for the recent chronic average glycemia [8]. The estimated glomerular filtration rate (eGFR) was calculated using the Chronic Kidney Disease Epidemiology Collaboration (CKD-EPI) equation  [18]. The N-terminal B-type natriuretic peptide (NT-proBNP) and cardiac troponin I (TnI) were dynamically monitored and peak values were recorded. The left ventricular ejection fraction (LVEF) was measured using the biplane Simpson method with echocardiography.

### Definitions and outcomes

In the present study, AMI was diagnosed based on the 4th universal definition of MI [19]. DM was defined as having a history of DM or newly diagnosed DM with HbA_1c_ ≥ 6.5%, fasting blood glucose ≥ 7.0 mmol/L, or 2-h plasma glucose of the oral glucose tolerance test (OGTT) ≥ 11.1 mmol/L [20]. Dyslipidemia was defined as low density lipoprotein cholesterol ≥ 3.4 mmol/L, high density lipoprotein cholesterol < 1.0 mmol/L, triglyceride ≥ 1.7 mmol/L or patients who were taking lipid-lowering medication [21]. Chronic kidney disease (CKD) was defined as renal structural abnormality or progressive functional loss lasting for > 3 months according to the Kidney Disease Improving Global Outcomes (KDIGO) criteria [22].

The primary endpoint of this study was acute kidney injury (AKI) defined as an increase in serum creatinine (Scr) of ≥ 0.3 mg/dL (26.5 μmol/L) or 1.5-fold higher than normal levels according to the Acute Kidney Injury Network (AKIN) classification [23]. The severity of AKI were classified as 3 stages based on the increased level in Scr (stage1: Scr elevated by 1.5–2 folds, stage2: 2–3 folds, stage3: ≥ 3 folds) [23]. If Scr concentration at first 72 h was lower than that at admission, the lower one would be considered as the basal concentration. The secondary endpoints included all-cause death and cardiogenic shock during hospitalization, representing the most severe hemodynamic consequences after an AMI. Cardiogenic shock was defined as prolonged hypotension, reduced cardiac output and decreased tissue perfusion with evidence of severe left ventricular dysfunction requiring IABP and/or inotropic agents [24].

### Statistical analysis

Continuous variables were expressed as mean ± standard deviation (SD) or median with interquartile range. Categorical variables were described as a number (n) with percentage (%). Differences were assessed using analysis of variance or Kruskal–Wallis H test for continuous variables and Pearson’s χ^2^ or Fisher’s exact test for categorical variables. The *p* for trend values were also calculated to show the significance of trends between SHR tertiles and event incidence. The logistic regression analysis was used to identify potential risk factors. The clinically relevant factors and unevenly distributed variables among groups were enrolled in the multivariate model, including age, gender, MI type (NSTEMI or STEMI), PCI treatment (with or without) and peak TnI values. Unadjusted and adjusted odds ratio (OR) with 95% confidence interval (CI) were calculated. The cut-off value of SHR for AKI prediction was identified with the maximum Youden index using a receiver operating characteristics (ROC) curve analysis. Discrimination was defined with areas under the curve (AUC), and AUC values were interpreted as follows: negligible (≤ 0.55), small (0.56–0.63), moderate (0.64–0.70) and strong (≥ 0.71) [25]. All tests were 2-tailed, and P < 0.05 was considered significant. The statistical analyses were performed using SPSS V.20.0 (SPSS Inc., Chicago, Illinois, USA).

## Results

### Baseline characteristics and in-hospital outcomes

**Fig. 1:**
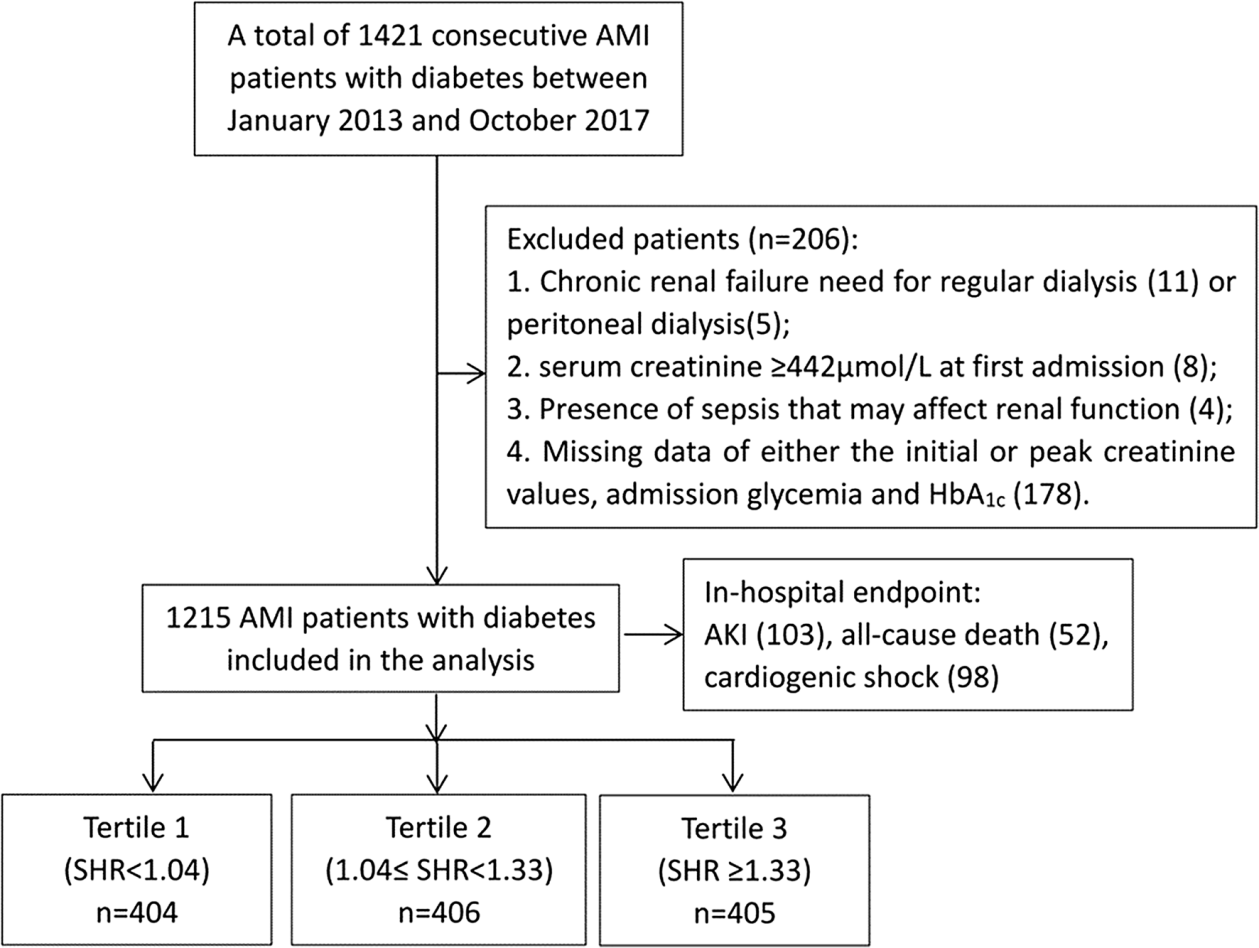
Flowchart of the study

Diabetic patients were divided according to the SHR tertile levels (Tertile 1: SHR < 1.04; Tertile 2: 1.04 ≤ SHR < 1.33; Tertile 3: SHR ≥ 1.33) (Fig. 1). As shown in Table 1, patients with higher SHR tertiles had higher frequent presence of STEMI, lower BMI, and higher peak values of NT-proBNP and TnI. They also had comparable LVEF level and similar values of hemoglobin, serum albumin, eGFR, low density lipoprotein cholesterol, and high-sensitivity C-reactive protein. There were no significant differences in age, gender, cardiovascular risk profile, in-hospital medication, rate of PCI treatment and usage of IABP. Overall, 103 (8.4%) developed AKI, 52 (4.2%) died, and 98 (8.0%) had cardiogenic shock during hospitalization. The rate of AKI (4.4%, 7.8%, 13.0%; *p* < 0.001), all-cause death (2.7%, 3.6%, 6.4%; *p* = 0.027) and cardiogenic shock (4.9%, 7.6%, 11.6%; *p* = 0.002) increased with the rising SHR tertiles. The tread analysis indicated a positive linear tread between event rate and SHR tertiles (all *p* for tread < 0.05) (Additional file 1: Table S1). Patients who developed AKI tended to have a more complicated AMI and a much poor prognosis. After multivariate adjustment, the occurrence of AKI was closely associated with an increased risk of all-cause death (OR = 4.68, 95%CI: 2.33–9.42, *p* < 0.001) and cardiogenic shock (OR = 5.52, 95%CI: 3.15–9.67, *p* < 0.001) (Additional file 1: Table S2).

**Table 1:** Baseline characteristics and clinical outcomes

	Tertile 1 (n = 404)	Tertile 2 (n = 406)	Tertile 3 (n = 405)	*p* value
Male, n(%)	273 (67.5%)	282 (69.4%)	273 (67.4%)	0.785
Age, years	66.2 ± 12.5	65.7 ± 11.8	65.9 ± 12.1	0.729
BMI, kg/m^2^	26.2 ± 3.7	26.9 ± 3.6	25.2 ± 3.6	0.002
STEMI, n(%)	160 (39.6%)	197 (48.5%)	227 (56.0%)	< 0.001
Cardiovascular risk factors				
Hypertension	291 (72.0%)	268 (66.0%)	288 (71.1%)	0.167
Dyslipidemia	197 (48.37%)	181 (44.5%)	181 (44.6%)	0.397
Previous MI	48 (11.8%)	46 (11.3%)	33 (8.1%)	0.173
Prior PCI	69 (17.0%)	70 (17.2%)	60 (14.8%)	0.580
CKD	49 (12.1%)	45 (11.0%)	63 (15.5%)	0.139
Smoking	235 (58.1%)	248 (61.0%)	224 (55.3%)	0.249
LVEF (%)	57.3 ± 10.8	56.4 ± 11.2	55.6 ± 11.3	0.114
Laboratory assessment				
ABG, mmol/L	8.25 ± 2.59	11.23 ± 3.32	15.18 ± 4.97	< 0.001
HbA_1c_, %	7.66 ± 1.72	7.58 ± 1.69	7.40 ± 1.55	< 0.001
Hemoglobin, g/L	126.9 ± 24.5	131.1 ± 23.7	129.6 ± 22.7	0.025
Albumin, g/dL	35.8 ± 4.8	34.9 ± 7.7	35.4 ± 5.9	0.921
eGFR, ml/(min*1.73m^2^)	74.6 ± 23.9	75.8 ± 24.0	72.1 ± 26.5	0.180
NT-proBNP, pg/mL	1653 (551, 5926)	1874 (653, 6114)	2302 (713, 7778)	0.036
Peak TnI, ng/mL	2.6 (0.5, 11.4)	5.7 (1.5, 22.2)	6.9 (1.6, 24.4)	< 0.001
hs-CRP, mg/L	6.8 (2.2, 14.8)	7.5 (1.9, 17.6)	7.7 (2.3, 20.6)	0.256
In-hospital medication				
Anti-platelet agents	387 (95.7%)	394 (97.0%)	389 (96.0%)	0.525
Statin	365 (90.3%)	364 (89.6%)	358 (88.3%)	0.657
ACEI or ARB	335 (82.9%)	342 (84.2%)	330 (81.4%)	0.436
Beta-blocker	323 (79.9%)	313 (77.0%)	311 (76.7%)	0.239
Diuretics	72 (17.8%)	64 (15.7%)	68 (16.7%)	0.665
PCI, n(%)	282 (69.8%)	304 (74.8%)	293 (72.3%)	0.272
IABP, n(%)	7 (1.7%)	13 (3.2%)	14 (3.4%)	0.276
In-hospital outcomes				
AKI	18 (4.4%)	32 (7.8%)	53 (13.0%)	< 0.001
All-cause death	11 (2.7%)	15 (3.6%)	26 (6.4%)	0.027
Cardiogenic shock	20 (4.9%)	31 (7.6%)	47 (11.6%)	0.002

Diabetic patients with AMI were divided according to the tertile levels of SHR (Tertile 1: SHR < 1.04; Tertile 2:1.04 ≤ SHR < 1.33; Tertile 3: SHR ≥ 1.33)

BMI: body mass index, STEMI: ST-segment elevation myocardial infarction, CKD: chronic kidney disease, PCI: percutaneous coronary intervention, LVEF: left ventricular ejection fraction, SHR: stress hyperglycemia ratio, ABG: admission blood glucose, HbA1c: glycated hemoglobin, eGFR: estimated glomerular filtration rate, NT-proBNP: N-terminal B-type natriuretic peptide, TnI: Troponin I, AKI: acute kidney injury

**Table 2:** Prognostic effect of SHR and ABG on the risk of in-hospital adverse events

	Univariate logistic analysis		Multivariate logistic analysis		ROC analysis
	OR (95% CI)	*P* value	OR (95% CI)	*P* value	AUC (95% CI)
AKI					
ABG	1.06 (1.02–1.10)	0.001	1.02 (0.89–1.13)	0.136	0.55 (0.48–0.61)
SHR	3.59 (2.31–5.58)	< 0.001	3.18 (1.99–5.09)	< 0.001	0.64 (0.58–0.69)*
All-cause death					
ABG	0.99 (0.93–1.05)	0.824	1.00 (0.94–1.06)	0.953	0.50 (0.42–0.57)
SHR	1.97 (1.14–3.42)	0.015	1.83 (1.03–3.23)	0.038	0.59 (0.51–0.66) *
Cardiogenic shock					
ABG	1.00 (0.95–1.04)	0.760	0.97 (0.92–1.03)	0.445	0.46 (0.39–0.52)
SHR	2.24 (1.45–3.46)	0.011	1.80 (1.12–2.87)	0.014	0.60 (0.54–0.66) *

OR was adjusted for age, gender, MI classification (STEMI or NSTEMI), PCI treatment (with or without) and peak TnI in the multivariate model. OR for per 1SD increase in ABG or SHR

ABG, admission blood glucose; SHR, stress hyperglycemia ratio; AKI, acute kidney injury; OR, odds ratio; CI, confidence interval

*Indicates a significant predictive value (*p* < 0.05) for the event

### Association between SHR level and AKI occurrence

**Fig. 2:**
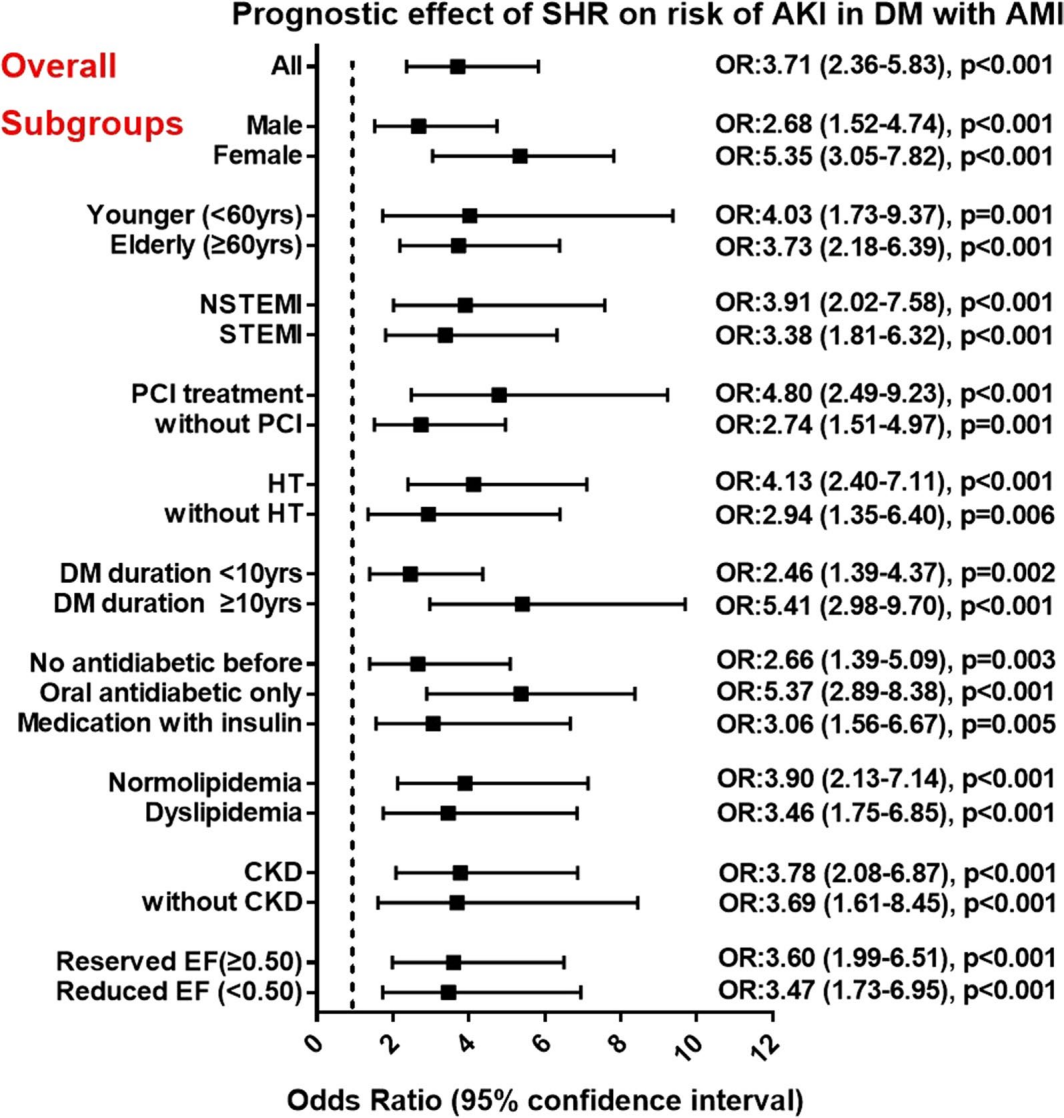
Relationship between SHR and the risk of AKI in subgroups of DM patients. Subgroup analysis for association between SHR and AKI occurrence in patients stratified by gender, age, MI classification, PCI treatment, hypertension, DM duration, treatment methods of DM, dyslipidemia, CKD, and LVEF level. Odds ratio (OR) was calculated by the univariate logistic regression analysis. OR for per 1 standard deviation increased in SHR. Vertical dotted line indicated the OR value of 1. NSTEMI: non-ST-segment elevation myocardial infarction, STEMI: ST-segment elevation myocardial infarction, PCI: percutaneous coronary intervention, HT: hypertension, DM: diabetes, CKD: chronic kidney disease, LVEF: left ventricular ejection fraction

The multivariate logistic regression analysis (Table 2) revealed that elevated SHR was strongly associated with the increased risks of AKI (OR = 3.18, 95%CI: 1.99–5.09, *p* < 0.001), all-cause death (OR = 1.83, 95%CI: 1.03–3.23, *p* = 0.038) and cardiogenic shock (OR = 1.80, 95%CI: 1.12–2.87, *p* = 0.014) among AMI patients with DM. Among the 103 identified AKI patients, the SHR level also increased in parallel with the degrees of Scr elevation (*p* = 0.039) (Additional file 1: Table S3), indicating a positive correlation between SHR and AKI severity. Meanwhile, ABG was no longer a risk factor of the adverse events after adjustment for major confounders. SHR remained a robust predictor of AKI in subsets of patients stratified by the age, gender, MI classification, PCI treatment, hypertension, dyslipidemia, CKD, LVEF level, diabetic duration and prior treatment for DM (all *p* < 0.05) (Fig. 2).

**Table 3:** Prognostic effect of the acute or stress hyperglycemia on AKI risk

AKI	Acute hyperglycemia (ABG ≥ 198 mg/dL)		Stress hyperglycemia (SHR ≥ 1.23)	
	OR (95% CI)	*P* value	OR (95% CI)	*P* value
Overall DM	1.60 (1.03–2.49)	0.036	2.43 (1.49–3.95)	< 0.001
History of DM				
Newly diagnosed	1.36 (0.54–3.42)	0.511	2.17 (1.04–5.13)	0.039
Known before	1.61 (0.94–2.75)	0.079	2.73 (1.53–4.84)	0.001
Duration of DM				
< 10 years	1.30 (0.70–2.39)	0.398	2.05 (1.05–4.01)	0.035
≥ 10 years	2.02 (0.98–4.15)	0.056	3.10 (1.45–6.63)	0.003
Prior treatment				
No medication	1.37 (0.65–2.91)	0.402	2.76 (1.13–4.95)	0.032
Oral antidiabetics	1.80 (0.77–4.21)	0.173	2.61 (1.06–6.42)	0.036
Insulin use	1.19 (0.51–2.75)	0.678	2.56 (1.06–6.19)	0.037

Multivariate logistic regression analysis for prognostic effect of acute or stress hyperglycemia on AKI risk in overall and subgroups of DM patients. Acute hyperglycemia was defined as ABG ≥ 198 mg/dL (11 mmol/L). Stress hyperglycemia was defined as SHR ≥ 1.23. This cut-off value of SHR was identified with maximum Youden index in all patients for AKI prediction using ROC analysis. Patients were stratified according to diabetic history, duration and prior treatment

OR was adjusted for age, gender, MI classification (STEMI or NSTEMI), PCI treatment (with or without) and peak TnI in the multivariate model

ABG, admission blood glucose; SHR, stress hyperglycemia ratio; DM, diabetes; AKI, acute kidney injury; OR, odds ratio; CI, confidence interval

Since SHR has been confirmed as a continuous risk factor for AKI, its association with the risk of AKI was further analyzed as a categorical variable. At ROC analysis, the cutoff value of SHR that maximized the sensitivity and specificity for AKI prediction in all patients was identified as 1.23. Totally, 44.5% of patients had a ratio above the cutoff (Additional file 1: Table S4). The incidence of AKI was 12.1% and 5.4% (*p* < 0.001) respectively in patients with SHR above and below the cutoff (adjusted OR 2.43, 95% CI: 1.49–3.95, *p* < 0.001). Further, patients were stratified based on the diabetic history, duration and prior treatment method. In each subgroup, the association between acute hyperglycemia defined as ABG ≥ 198 mg/dL and AKI risk was not significant, whereas stress hyperglycemia with SHR ≥ 1.23 was significantly associated with an increased risk of AKI (all *p* < 0.05) (Table 3). This result indicated that the novel index SHR had a superior predictive value for AKI occurrence, and this ability was not influenced by critical relevant factors for DM.

**Table 4:** Logistic analysis of potential clinical risk factors for AKI

Variables	Univariate logistic analysis		Multivariate logistic analysis	
	OR (95% CI)	*p* value	OR (95% CI)	*p* value
Female	1.15 (0.74–1.79)	0.530	NA	…
BMI	1.02 (0.96–1.08)	0.469	NA	…
Hypertension	0.88 (0.56–1.38)	0.587	NA	…
Dyslipidemia	0.76 (0.50–1.17)	0.222	NA	…
Previous MI	1.01 (0.51–1.99)	0.981	NA	…
Prior PCI	0.95 (0.53–1.69)	0.872	NA	…
Smoking	0.94 (0.61–1.43)	0.782	NA	…
CKD	2.78 (1.54–4.99)	0.001	1.07 (0.69–1.44)	0.240
Emergent angiology	1.19 (0.76–1.86)	0.447	NA	…
Hemoglobin	0.98 (0.97–0.99)	0.002	1.00 (0.98–1.01)	0.960
Albumin	0.97 (0.95–1.01)	0.134	NA	…
LVEF	0.94 (0.93–0.96)	< 0.001	0.96 (0.93–0.99)	0.012
ln (NT-proBNP)	1.39 (1.22–1.58)	< 0.001	1.13 (1.05–1.22)	0.008
Peak TnI	1.02 (1.01–1.03)	0.015	1.01 (0.99–1.02)	0.223
eGFR	0.95 (0.94–0.96)	< 0.001	0.94 (0.92–0.96)	< 0.001
hs-CRP	1.04 (1.02–1.06)	< 0.001	1.02 (0.99–1.04)	0.151
SHR	3.71 (2.36–5.83)	< 0.001	2.74 (1.50–4.99)	0.001

Statistically significant variables with univariate analysis were enrolled in the multivariate model. Odds ratio (OR) for per 1 standard deviation increased in each continuous variable. N-terminal B-type natriuretic peptide (NT-proBNP) was natural logarithmically transformed to ln (NT-proBNP)

NA: not assessed, CI: confidence interval, BMI: body mass index, MI: myocardial infarction, PCI: percutaneous coronary intervention, CKD: chronic kidney disease, LVEF: left ventricular ejection fraction, TnI: Troponin I, eGFR: estimated glomerular filtration rate, hs-CRP: high-sensitivity C-reactive protein, SHR: stress hyperglycemia ratio

**Fig. 3:**
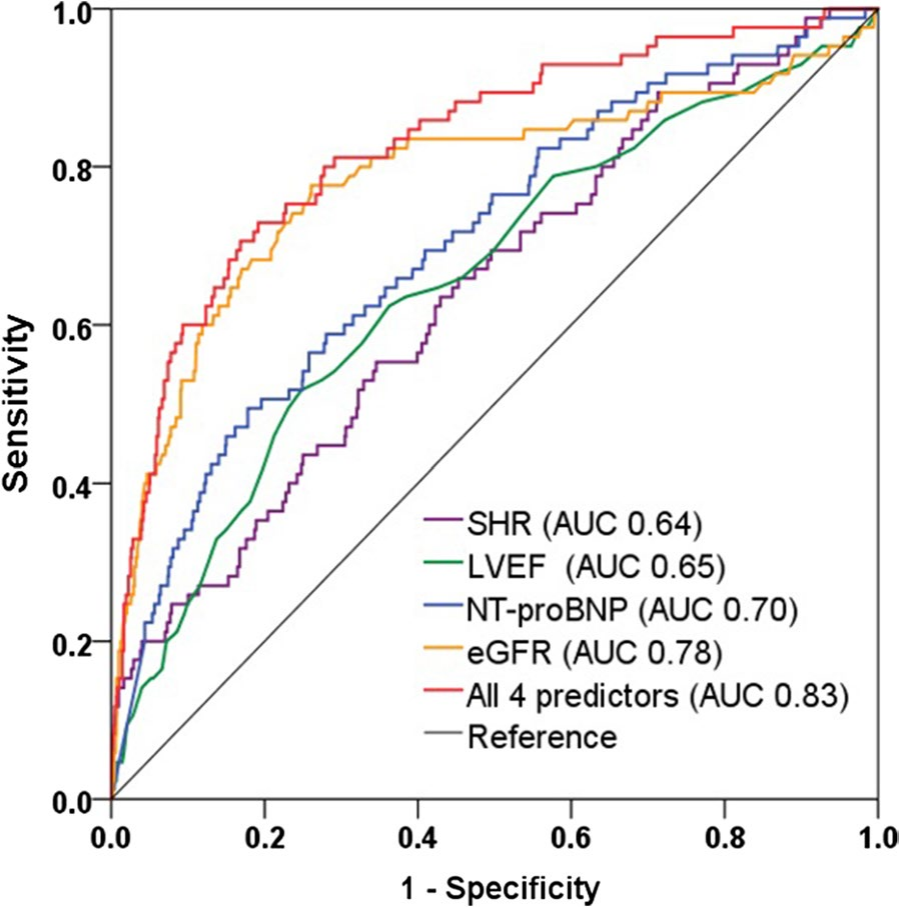
Predictive value of SHR and other predictors for AKI in DM patients. Receiver-operating characteristic curves showing the predictive value of SHR, other predictors, and the combined risk model in diabetic patients. SHR: stress hyperglycemia ratio, NT-proBNP: N-terminal B-type natriuretic peptide, LVEF: left ventricular ejection fraction, eGFR: estimated glomerular filtration rate, AUC: area under the curve

Apart from SHR, the other independent predictors of AKI were identified, including eGFR, LVEF and NT-proBNP (Table 4). The ROC analysis indicated that the discriminatory power of SHR for AKI prediction was moderate (AUC 0.64), while a risk model consisted of these 4 predictors yielded a good predictive value (AUC 0.83) for AKI in diabetic patients (Fig. 3).

## Discussion

In the present study, we found that SHR, instead of ABG, was significantly associated with the risk of AKI and adverse CV events in AMI patients with DM, even after adjustment for major confounders. The predictive value of SHR for AKI was better than admission glycemia alone, indicating that the combined evaluation of acute and chronic glycemic levels enables more accurate prediction of AKI after an AMI, especially in patients with DM.

The concept of stress hyperglycemia emphasizes a relative acute increase of glycemia in response to stress reaction or critical illness [26]. Recently, stress hyperglycemia is increasingly being seen in AMI patients with or without DM and has been recognized as one of the critical risk factors for AKI and adverse complications [27]. However, the association between elevated glycemia at admission and risk of AKI was more prominent in non-DM patients than in DM after AMI [28]. Several studies proved that admission glycemia (ABG) could predict AKI and CV events in non-DM [29, 30], but it was no longer a significant predictor of prognosis in DM [9, 10]. Similarly, we found that elevated ABG was not a risk factor for AKI and CV events in AMI patients with DM. The explanation might be that a single ABG value did not take the average glycemic level into account. Many diabetic patients had achieved a good glycemic control after receiving an optimal glucose-lowering treatment while others had not. Thus, a high level of ABG did not necessarily reflect a genuine glycemic rise in response to AMI, especially in diabetic patients with chronically elevated glycemic levels, and the performance of ABG for AKI prediction might be attenuated in DM. To address this issue, a more refined marker is needed to identify the true stress hyperglycemia and to facilitate the discrimination of AKI.

The index SHR, also known as acute-to-chronic glycemic ratio, was first introduced in 2015 through quantifying the magnitude of a relative glycemic rise from chronic glycemia of the past 2–3 months [8]. As proposed by Roberts, SHR could identify true stress hyperglycemia and was a better biomarker of critical illness than admission glycemia in patients across the whole glycemic spectrum [8]. Since then, several studies have confirmed the prognostic power of SHR in AMI patients and in all-comers undergoing PCI [12–14]. Our recent study also proved that SHR was more accurate in predicting in-hospital morbidity and mortality than admission glycemia alone among diabetic STEMI patients after PCI [15]. In line with previous studies, we found that SHR was more associated with the risk of AKI than ABG, and its level was also correlated with the AKI severity. Stress hyperglycemia defined by SHR ≥ 1.23 was a strong predictor of AKI in overall and in subgroups of patients. These data suggest that this novel biomarker may provide more prognostic information on AKI in AMI patients with DM.

A large number of studies have identified various risk factors of AKI occurrence in AMI, including advanced age, hypertension, DM, CKD, anemia, severe Killip class, tachycardia at presentation, longer reperfusion time, decreased serum albumin and more contrast used [31–33]. In the present study, we found that higher SHR and baseline cardiorenal dysfunction (reduced eGFR, decreased LVEF and increased NT-proBNP) were independent predictors of AKI. A combined model of SHR, LVEF, NT-proBNP, and eGFR showed a satisfactory discrimination for AKI, which may assist in the early detection of AKI in diabetic patients after an AMI.

A strong relationship among stress hyperglycemia, AKI occurrence and poor prognosis in patients with AMI has been identified for decades. Our cohort indicated that AMI patients with DM who had higher SHR tertiles were more likely to develop AKI, cardiogenic shock and all-cause death. The underlying mechanisms are manifold. Beyond the widely recognized hemodynamic influences of reduced cardiac output and venous congestion that contribute to hypoperfusion of the kidneys and a marked decline in renal function, an abnormal burst of neuroendocrine and inflammatory activation also accelerates the renal injury [34, 35]. Of these, stress hyperglycemia plays a pivotal role in the AKI development followed by AMI. An acute glycemic rise can induce osmotic diuresis and thus lead to volume depletion and dehydration. Moreover, acute hyperglycemia directly enhances inflammation and oxidative stress [36], which may further suppress the flow-mediated vasodilation and reduce renal perfusion [37]. All these pathophysiologic changes in response to acute hyperglycemia could markedly exacerbate the deleterious effects of contrast agents, IABP, and other contributing factors on the kidneys and finally result in a poor prognosis.

In clinical practice, the dynamic measurements of Scr should be emphasized because daily Scr value and its change pattern facilitate to identify AKI at early stages and they are stronger predictors of in-hospital mortality than the initial Scr only [38]. Meanwhile, stress hyperglycemia and AKI are both commonly seen complications after AMI. The AKI occurrence represents the confluence of cardiorenal interactions, involving the cross-talk between the failing heart and acute responses of kidneys, whereas stress hyperglycemia is implicated in hemodynamic instability, neurohormonal release and inflammatory reaction, thereby exerting detrimental effects on renal function [35]. Due to the good performance of SHR for AKI prediction, it may be used as a bedside marker to early discriminate patients at high risks to develop AKI. Moreover, the assessment of SHR may help practitioners to tailor glucose-lowering strategies. Till now, the tight glycemic control strategies are proved to have neutral or even deleterious effects on CV outcomes in DM patients [39–41]. Not only is that an intensive glucose lowering can markedly increase the risk of severe hypoglycemia, [42] but the phenomenon of metabolic glucose memory also exists in DM, indicating that the prior glycemic control may have a sustained effect that persists even after returning to the current glycemic status [43]. Therefore, a relative glycemic rise rather than admission glycemia alone should be more emphasized. For patients who had a high level of glycemia at admission, chronic glycemic levels still need to be considered to set the optimal target value of glucose lowering. Meanwhile, for patients whose glucose levels are below the conventional treatment threshold (11 mmol/L), SHR is useful to discriminate a real glycemia rise and thus may assist physicians to decide when to initiate glucose-lowering therapy. The prospect of the SHR would be promising for its good effectiveness and applicability. But far from claiming superiority or perfection, we should note that the predictive accuracy of this novel marker is still moderate and its performance should to be further verified by external validation.

### Limitations

Some limitations should be mentioned. First, despite major clinically relevant variables were adjusted in the multivariate model and subgroup analysis was performed, the effects of nephrotoxic drugs and some other possibly residual confounding factors on AKI development were not analyzed, which may affect the outcomes. Second, similar with other studies on AKI, the time interval during measurements of serum creatinine at admission and the first 72 h was not predetermined and fixed. This may add bias to the identification of acute increase in serum creatinine. Third, this was an observational study at a single center, the sample size was limited and a definite cause-effect relationship between stress hyperglycemia and AKI cannot be established. Further multicenter and larger randomized controlled trials are needed to validate our findings, to identify more specific biomarkers of stress hyperglycemia for AKI prediction, and to investigate whether a glucose-lowering strategy targeted on SHR instead of ABG may result in a renal-protective effect in AMI patients with DM.

## Conclusions

In AMI patients with DM, the novel glycemic marker SHR was more closely associated with the risk of AKI, in-hospital mortality and cardiogenic shock than admission glycemia alone. The assessment of SHR may help practitioners to identify true stress hyperglycemia, to discriminate high-risk patients of AKI at early stage, and to facilitate the pre-emptive decision making on glucose-lowering treatment and renal-protective strategies.

## Supplementary Information

**Additional file 1.** **Table S1.** Trend analysis among SHR tertile levels. **Table S2.** Association between AKI and in-hospital CV outcomes. **Table S3.** Association between SHR level and AKI severity. **Table S4.** Prognostic effect of the identified cut-off value of SHR for AKI.

## Data Availability

The datasets used and/or analyzed during the current study are available from the corresponding author on reasonable request.

## Abbreviations

AMI: Acute myocardial infarction
DM: Diabetes
AKI: Acute kidney injury
PCI: Percutaneous coronary intervention
SHR: Stress hyperglycemia ratio
ABG: Admission blood glucose
HbA_1c_: Glycated hemoglobin
eAG: Estimated average glucose
LVEF: Left ventricular ejection fraction
NT-proBNP: N-terminal B-type natriuretic peptide
eGFR: Estimated glomerular filtration rate
