# Supplementary material for: Predictive value of stress hyperglycemia ratio for the occurrence of acute kidney injury in acute myocardial infarction patients with diabetes
Source: BMC Cardiovasc Disord. 2021 Mar 30;21:157. doi: 10.1186/s12872-021-01962-2 (PMC8008672; doi:10.1186/s12872-021-01962-2)
Supplement: Supplementary file 1 — Additional file 1. Table S1. Trend analysis among SHR tertile levels. Table S2. Association between AKI and in-hospital CV outcomes. Table S3. Association between SHR level and AKI severity. Table S4. Prognostic effect of the identified cut-off value of SHR for AKI. [file 12872_2021_1962_MOESM1_ESM.docx]

**Supplementary Materials of the Manuscript**

**Predictive value of stress hyperglycemia ratio for the occurrence of acute kidney injury in acute myocardial infarction patients with diabetes**

**Short title**: SHR for AKI prediction in AMI with DM

Side Gao^1,2^; Qingbo Liu^1^; Hui Chen^1^; Mengyue Yu^2*^; Hongwei Li^1*^

^1^Department of Cardiology, Cardiovascular Center, Beijing Friendship Hospital, Capital Medical University, Beijing, China;

^2^Department of Cardiology, Fuwai Hospital, National Center for Cardiovascular Diseases, Chinese Academy of Medical Sciences and Peking Union Medical College, Beijing, China.

Correspondence to:

Mengyue Yu, MD, PhD, FACC, FESC, Department of Cardiology, Fuwai Hospital, National Center for Cardiovascular Diseases, Chinese Academy of Medical Sciences and Peking Union Medical College, Bei Li Shi Road 167, Xicheng District, Beijing, 100037, China. Email: [yumy73@163.com](mailto:yumy73@163.com).

Hongwei Li, MD, PhD, FACC, FESC, Department of Cardiology, Cardiovascular Center, Beijing Friendship Hospital, Capital Medical University, Yongan Road 95, Xicheng District, Beijing, 100050, China. Email: [lhw19656@sina.com](mailto:lhw19656@sina.com).

**Supplementary Table 1 Trend analysis among SHR tertile levels**

|  | Tertile 1  (n=404) | Tertile 2  (n=406) | Tertile 3  (n=405) | P value | P for trend |
| --- | --- | --- | --- | --- | --- |
| STEMI, n(%) | 160 (39.6%) | 197 (48.5%) | 227 (56.0%) | <0.001 | <0.001 |
| NT-proBNP, pg/mL | 1653 (551, 5926) | 1874 (653, 6114) | 2302 (713, 7778) | 0.036 | 0.048 |
| Peak TnI, ng/mL | 2.6 (0.5, 11.4) | 5.7 (1.5, 22.2) | 6.9 (1.6, 24.4) | <0.001 | <0.001 |
| In-hospital outcomes |  |  |  |  |  |
| AKI | 18 (4.4%) | 32 (7.8%) | 53 (13.0%) | <0.001 | <0.001 |
| All-cause death | 11 (2.7%) | 15 (3.6%) | 26 (6.4%) | 0.027 | 0.009 |
| Cardiogenic shock | 20 (4.9%) | 31 (7.6%) | 47 (11.6%) | 0.002 | 0.001 |

Clinically relevant variables that were unevenly distributed across SHR tertiles (presence of STEMI, NT-proBNP and peak TnI) and in-hospital outcomes were again presented and the P for trend values among 3 groups were calculated. Diabetic patients with AMI were divided according to the tertile levels of SHR (Tertile 1: SHR<1.04; Tertile 2:1.04≤SHR<1.33; Tertile 3: SHR≥1.33). STEMI: ST-segment elevation myocardial infarction, TnI: Troponin I, AKI: acute kidney injury.

**Supplementary Table 2 Association between AKI and in-hospital CV outcomes**

|  | Univariate logistic analysis | | Multivariate logistic analysis | |
| --- | --- | --- | --- | --- |
|  | OR (95% CI) | P value | OR (95% CI) | P value |
| All-cause death |  |  |  |  |
| Without AKI | 1 (reference) | … | 1 (reference) | … |
| AKI | 7.46 (4.03-13.82) | <0.001 | 4.68 (2.33-9.42) | <0.001 |
| Cardiogenic shock |  |  |  |  |
| Without AKI | 1 (reference) | … | 1 (reference) | … |
| AKI | 7.14 (4.34-11.74) | <0.001 | 5.52 (3.15-9.67) | <0.001 |

Logistic regression analysis for association between AKI and the risk of in-hospital all-cause death and cardiogenic shock. Odds ratio (OR) was adjusted for age, gender, MI classification (STEMI or NSTEMI), PCI treatment (with or without) and peak TnI in the multivariate model. AKI: acute kidney injury, CI: confidence interval.

**Supplementary Table 3 Association between SHR level and AKI severity**

| AKI patients  (n=103) | AKI Stage1  (n=53) | AKI Stage2  (n=31) | AKI Stage3  (n=19) | P value |
| --- | --- | --- | --- | --- |
| SHR level | 1.39±0.50 | 1.50±0.76 | 1.72±0.58 | 0.039 |

The classification of AKI severity was defined according to the increased level of serum creatinine (Scr) in line with the AKI network (Stage1: Scr↑ of 1.5-2 folds, Stage2: Scr↑ of 2-3 folds, Stage3: Scr↑ of ≥3 folds). SHR: stress hyperglycemia ratio, AKI: acute kidney injury.

**Supplementary Table 4 Prognostic effect of the identified cut-off value of SHR for AKI.**

|  | Incidence of AKI | | Univariate logistic analysis | | Multivariate logistic analysis | |
| --- | --- | --- | --- | --- | --- | --- |
|  | n/N (%) | P value | OR (95% CI) | P value | OR (95% CI) | P value |
| SHR <1.23 | 37/674 (5.4%) | … | 1 (reference) | … | 1 (reference) | … |
| SHR ≥1.23 | 66/541(12.1%) | <0.001 | 2.39 (1.54-3.69) | <0.001 | 2.43 (1.49-3.95) | <0.001 |

Pearson’s χ^2^ test and logistic regression analysis for incidence and risk of AKI in overall with SHR above the cut-off value (≥1.23). This value was identified with maximum Youden index in all AMI patients with DM for AKI prediction. Odds ratio (OR) was adjusted for age, gender, MI classification (STEMI or NSTEMI), PCI treatment (with or without) and peak TnI in the multivariate model. SHR: stress hyperglycemia ratio, AKI: acute kidney injury, DM: diabetes, CI: confidence interval.
